# Perception of parents about second hand smoke on the health of their children: an ethnographic study

**DOI:** 10.1016/j.rpped.2015.02.003

**Published:** 2015

**Authors:** Fabiane Alves de Carvalho Ribeiro, Micaele Kedma Ribeiro de Moraes, Joyce Cristina de Morais Caixeta, Jullieth Nadja da Silva, Amanda Sanches Lima, Samara Lamounier Santana Parreira, Viviane Lemos Silva Fernandes

**Affiliations:** a Centro Universitário de Anápolis (UniEvangélica), Anápolis, GO, Brazil

**Keywords:** Pollution for tobacco smoke, Parents, Child

## Abstract

**Objective:**

To analyze the perception of parents about secondhand smoking in their children's health.

**Methods:**

Ethnographic qualitative and quantitative study. We sought the point of view and understanding of the parents who were active smokers in relation to environmental tobacco smoke (ETS) and secondhand smoking. Mothers and fathers who are active smokers and that live with their children from seven different public schools in the city of Anápolis, Midwest Brazil, were interviewed in the first semester of in a reserved room in the schools. A descriptive and qualitative analysis was carried out through the ethnography.

**Results:**

58 parents with an average time of smoking of 15.3 years and an average quantity of cigarettes smoked per day of 2 were interviewed. Among them, 59% did not know what ETS was, and 60% stated knowing what a secondhand smoker was. However, when questioned about their children as secondhand smokers, 52% did not consider them to be. Some parents knew some of the effects of secondhand smoking in the health of their children. However, the majority (52%) of them did not believe that their children would suffer any respiratory impairment or did not know about these impairments.

**Conclusions:**

Children were exposed to environmental tobacco pollution in their residence if one considers parental duration of smoking and average of cigarettes smoked per day. There was a lack of knowledge of the parents about ETS, secondhand smoking and the evils that cigarettes could cause in the health of their children.

## Introduction

Second-hand smoke is defined as the inhalation of smoke from tobacco products by nonsmokers who live indoors with smokers, being the 3rd leading cause of preventable death worldwide, after active smoking and excess drinking. The smoke of tobacco products indoors is called environmental tobacco smoke (ETS), consisting in more than four thousand components, with more than 40 carcinogens. According to the World Health Organization (WHO), ETS becomes more damaging indoors, as polluted air can contain up to three times more nicotine and carbon monoxide and 50 times more carcinogens than the smoke that goes through the cigarette filter, inhaled by the active smoker.^1^^-^ 3

Data from the National Cancer Institute (INCA) (2011) point out that the Brazilian Unified Health System (SUS) and Social Security annually spend approximately R$ 37 million on diseases and deaths caused by passive smoking, and that the number of deaths is approximately 3000 nonsmokers per year. Moreover, according to INCA, the World Health Organization (WHO) states that each year 5 million individuals die due to smoking-related diseases and that smoking is the main preventable cause of morbidity and mortality. It is estimated that 1100 individuals a day die due to smoking. Studies show that there are approximately 1.2 billion smokers worldwide, with 24.6 million of them in Brazil alone. WHO estimates report that 40% of children worldwide are exposed to tobacco smoke.^4^^-^ 6

Smoking affects smokers and nonsmokers and, in the long-term, results in deleterious effects on the body such as increased risk of cancer in the respiratory, digestive and urinary tracts, pancreas and cervix, risk of coronary heart diseases and stroke. Exposure to ETS is associated with several diseases. Children exposed to ETS are more frequently affected by middle ear infections, reduced lung function, respiratory diseases such as pneumonia, bronchitis and asthma exacerbations. Babies exposed to ETS have a risk five-fold higher of developing sudden infant death syndrome, and have higher risk of pulmonary diseases in the first year of life.^1^^,^ 7 ^-^ 12 The absorption of cigarette smoke by children living with smoking parents indoors may differ in their concentration, according to the number of smokers in the household and the number of cigarettes smoked to which the child is exposed. The WHO reports that the risks caused by second-hand smoking to health are significant, being well established and preventable.^1^^,^ 13

Studies report parental knowledge about passive smoking; however, the lack of information is still evident among the lower socioeconomic classes. Nevertheless, even among those who know about the effects of ETS, there are parents who expose their children to the harmful effects of ETS at home.^13^^,^ 14 Parents should understand that any exposure to tobacco should be considered a risk factor for several diseases. Smoking control measures that promote the maintenance of a smoking-free lifestyle among children of all ages should be implemented and encouraged. Thus, this study aimed to analyze the perception of parents regarding the effect of passive smoking on the health of their children.

## Method

This is a quali-quantitative study. Authorization was requested from all involved schools to develop the study. The study was approved by the Institutional Review Board (protocol: 161,431) of Centro Universitário de Anápolis and was carried out in seven public schools in Anapolis, Goias state, BR. Contact with the parents was made at school meetings, when current smokers were identified through a previous dialog; thus, participants were informed about the study and invited to participate. Of the 356 parents identified in all schools, 96 reported being smokers, and 60 (63%) fathers or mothers agreed to participate and signed the Informed Consent form; the sample included only one smoker from each household or both. After consent, parents answered the questions of the proposed interview. Only fathers or mothers who were active smokers and lived daily with their children aged 6-12 years were included in the study, totaling 58 parents who smoked. The two other parents were excluded because they did not answer all of the questions in the interview. Sample size was attained by applying the data saturation criterion.

The interviews were carried out according to a protocol that included data on gender, age, duration of smoking, child's time of exposure to ETS, family income and level of education. The guiding questions were outlined for this research and consisted of the following: do you know what a passive smoker is? Do you consider your child a passive smoker? Do you think that your child, when in the same room with you when you are smoking, may have some damage to his/her health? Do you know what type of damage this is? Were you aware of the influence of passive smoking on respiratory health (lungs) of your child? What do you think might happen? Do you know what environmental tobacco smoke is? Have you, at any point, been advised on the influence of passive smoking on the health of your children?

Interviews were carried out in the first half of 2014, in a private room, available at school, started and recorded only when the participants felt comfortable to do so. At the end of the interview, parents were instructed about the topic, in order to answer questions aiming to alert them about the several effects and harms of ETS and passive smoking on the health of their children.

The interviews were transcribed. At this stage, the recorded content was carefully played and listened to, with the answers of all parents being accurately transcribed, in the form of narrative.

Qualitative data were analyzed through careful reading, seeking to capture the significant aspects of the narratives, focusing on the words or the senses. After extracting the categories of analysis of the studied phenomenon, the most remarkable and similar phrases were then selected to formulate categories of analysis of the ethnographic results. According to Rosa, Lucena and Crossetti, ethnography is currently being used as an important method to enhance facts related to individuals’ lifestyles, considering the physical, cultural, social and environmental aspects and the way these factors influence their life conditions, based on the respondents.^15^^,^ 16 By employing ethnography, it was possible to outline a trajectory to understand and interpret the experience of active smokers and their perceptions about the effects of passive smoking on their children's health.

Regarding the variables - gender, age, duration of smoking, number of cigarettes smoked a day, time of children's exposure to ETS, family income and educational level of the parents, the descriptive analysis of data was carried out as mean, standard deviation and relative and absolute frequencies.

## Results

The study sample consisted of 58 individuals, 66% females and 34% males, all active smokers with a mean age of 30 years. The 58 adults lived with 95 children, with a mean age of 9.2±1.7 years, 56% of which were females.

When parents were asked about duration of smoking, they reported a mean time of 15.3 years. Regarding the number of cigarettes smoked a day and duration of children's exposure to tobacco smoke, the mean was 20.1 cigarettes/day and around 2.8 h of exposure to ETS a day. When analyzing the knowledge of these parents about the ETS, 59% said they had no knowledge of the subject; however, 60% of parents reported knowing what a passive smoker was, while 52% said they did not consider their child a passive smoker. The mean family income was around R$ 1389.00; as for the level of education, 45% said they had finished Elementary School; 40% had finished High School; 8% had attended College/University, and 7% were illiterate.

The generated data based on narratives and descriptions constituted the analysis content. A detailed reading of responses aimed to capture the presence of significant aspects contained in the participants’ statements. The most remarkable and similar phrases among the parents were chosen for each question.

When asked "*Do you know what a passive smoker is?*", most parents, 60%, said they did; however, when analyzing the answers, we observed a poor level of knowledge among parents, as shown by the following answers:

"*Yes. When I’m smoking and you can smell it.*"

"*Yes, when I’m smoking and a person is close to me.*"

"*Yes, when you smoke without protective filter.*"

"*Yes, people who are close to those who are smoking.*"

"*Yes, it is the individual who is in the same environment as the smoker.*"

When they were asked "*Do you consider your child a passive smoker?"*the majority of parents, 52%, said they did not. Only two parents justified the reason for that with the following answers:

"*No, I do not smoke near them.*"

"*No, because I smoke far from them.*"

The descriptive analysis of the answers to the questions "*Were you aware of the influence of passive smoking on respiratory health (lungs) of your child? What do you think might happen?*" showed that some parents had knowledge about the influence of passive smoking on the respiratory health of their children, as shown by the answers:

"*Yes, I think it can cause bronchitis, I do not know, it somehow affects the lung.*"

"*Yes, you can develop several types of lung disease.*"

"*It goes into the lung, doesn’t it? And eventually causes a lung problem.*"

"*Yes, health problems in general, it can lead to respiratory diseases.*"

"*Yes, you can have lung problems.*"

"*I think you have problems, but I do not know exactly what can happen.*"

However, it was observed, in the descriptive analysis that the majority of parents, 59%, reported not knowing what ETS is and, considering the analysis of the answers to the same question above, it was possible to confirm the lack of information on the subject. Most had similar answers, not believing that their children could suffer some respiratory impairment, as demonstrated by the answers below:

"*No, they hardly stay next to me and when I smoke, I stay away*."

"*My husband and I try not to smoke near the boys, so I think they will have no problems*."

"*I think nothing will happen to the lungs. I think they may have other problems, but not in the lungs*."

"*In their lungs I don’t think so, because it is my lung that suffers*."

"*No, I think only my husband and I are the ones who will have some kind of problem.*"

"*No, I see only in cigarettes packs, but we do not think that can happen to us.*"

When asked if they, at any time, had been given instructions about the influence of passive smoking on their children's health, most parents said they had never received any guidance, with the following similar answers:

"*No, I had never heard of this subject.*"

"*No, no one ever told me anything.*"

"*No, I had never heard of second-hand smoke.*"

"*No. This is the first time I hear something about it.*"

## Discussion

Second-hand smoke is the secondary exposure to cigarette smoke or other tobacco products by nonsmokers who live with smokers indoors. The concern regarding the effect of second-hand smoke on children is on a larger scale, because their bodily systems are still developing, especially the immature respiratory system, which can be more sensitive to such exposure.^1^^,^ 17 ^-^ 19

The environment where the child lives, as well as living with adult individuals, can exercise influence on their development. When analyzing the results of this study in relation to parental smoking time, we observed a mean of 15.3 years. Similarly, a study that assessed the prevalence of respiratory symptoms in children and adolescents with 174 parents pointed out that, regarding the duration of smoking, 46.5% of mothers and 57.9% of fathers had smoked for 14 years or longer. A strong association was also demonstrated between exposure to household smoking and the development and increased severity of asthma in children.^18^^-^ 20

As for the number of cigarettes smoked daily, in this study the parents smoked on average 20.1 cigarettes a day. A longitudinal study evaluating the increased incidence of asthma in children of smoking mothers showed that children whose mothers smoked more than half a pack of cigarettes a day, especially in the first two years of life, were about twice as likely to develop asthma, and this fact might be associated with the greater contact between mother and child in this stage of childhood.^21^ In this study, the mean time of exposure of children to passive smoking was 2.83 h a day. Venners et al. evaluated 1718 children and adolescents and reported a mild association between parental smoking and the decline in lung function of children who were passive smokers.^22^

Exposure to ETS is associated with high morbidity and mortality in younger children. Children's health is especially vulnerable to the risk of such exposure, including upper and lower respiratory tract infections. A study that evaluated the child's exposure to tobacco smoke and its association with asthma development showed that oxidative/antioxidant balance strongly leaned to the oxidative side in preschoolers who were passive smokers, with the development of acute and chronic ear infections, asthma exacerbation, neurodevelopmental alterations, behavioral problems and decreased school performance. The free radicals originated from cigarette smoke are considered a main cause of atherosclerosis and cancer and have the capacity to directly and indirectly induce oxidative stress.^19^^,^ 23

A study carried out with children with asthma symptoms showed that 60% of the parents had less than 5 years of schooling. A research that analyzed the differences in the prevalence of smokers among socioeconomic groups found that among illiterate men or those with less than 4 years of schooling, smoking prevalence reached 48.6%. Other studies have also demonstrated that households where parents had lower educational level had a higher occurrence of smoking.^24^^-^ 28

When considering family income, this study showed a mean of approximately two minimum wages. Studies considering the population stratification by income and occupation, showed increased consumption of cigarettes, two to three times higher, in groups with worse social and economic status. It is important to question why parents with lower socioeconomic status are the ones who smoke the most. Smoking may be a response to the stress and difficulties associated with living in an economically deprived environment.^29^

When parents were asked whether they were aware of the effect of passive smoking on their children's respiratory health, the majority answered that they did not think their children would have any problems, not even regarding respiratory health. Other parents reported they believe that the children might only have respiratory problems, showing that parents have a lack of information about the consequences that cigarette smoke can bring to their children's health, often victimized by the ignorance and neglect of adult smokers. Studies have shown that tobacco smoke generates direct and indirect impacts on the child's overall health and some of them, children of smoking parents, showed factors associated with learning and language difficulties and behavioral problems.^30^ In the present study, some parents reported they did not have any information about passive smoking. If the information does not reach the poorest and least informed layers of a society, children with low socioeconomic status will be more vulnerable to the deleterious effects of ETS. As a result, these children are more likely to become active smokers and acquire respiratory diseases, continuing the tobacco family cycle.^31^

Efforts to prevent morbidity and premature mortality depend on prevention programs, protection policies against tobacco, against tobacco exposure and effective smoking cessation programs. The cessation helps to reduce the burden of diseases caused by smoking, due to the immediate benefits for the health of smokers and people who live with smokers. However, for many smokers, smoking cessation remains a distant goal. The change in behavior may occur when the motivation for cessation is altered, because there is a common ignorance about the magnitude of tobacco damage, combined with the tendency of smokers to underestimate their personal risk. The strategy of approaching parents with the promotion of health of children exposed to tobacco smoke, instead of personal risk, can be particularly effective when the smoker believes that the health of the child will have several benefits.^32^^,^ 33

Parents and teachers are role models during childhood. Parents who smoke are strong examples for their children to become smokers, which will not only make them passive smokers, but can also influence them to start smoking even at young ages, causing them severe health problems. It is important to develop actions that will lead the family and the school to create preventive actions related to tobacco consumption.

It is worth mentioning the difficulty of recruiting parents to voluntarily participate in the study or attend the school meetings, just as there was a significant limitation in other schools to open their doors to this type of approach, which eventually limited sample size. A significant weakness of this study is the fact that it represents a specific population limited to a geographical region (seven public schools in the city of Anapolis, state of Goias), of which results do not necessarily apply to other regions of the country; however, it indicates the need for more research exploring parental perception, as the scarcity of publications on the subject was observed.

We conclude that children are exposed to environmental tobacco smoke in the households, which was made evident by the data, duration of smoking and mean number of cigarettes smoked a day. However, a lack of knowledge on the part of parents regarding environmental tobacco smoke, passive smoking and the adverse effects that smoking can have on their children's health was observed.
