# Supplementary material for: Perception of parents about second hand smoke on the health of their children: an ethnographic study
Source: Rev Paul Pediatr. 2015 Oct-Dec;33(4):394–9. doi: 10.1016/j.rpped.2015.02.003 (PMC4685558; doi:10.1016/j.rpped.2015.02.003)
Supplement: Translation

# Translation: Percepção dos pais a respeito do tabagismo passivo na saúde de seus filhos: um estudo etnográfico

**Authors:** Fabiane Alves de Carvalho Ribeiro, Micaele Kedma Ribeiro de Moraes, Joyce Cristina de Morais Caixeta, Jullieth Nadja da Silva, Amanda Sanches Lima, Samara Lamounier Santana Parreira, Viviane Lemos Silva Fernandes

**Affiliations:** a Centro Universitário de Anápolis (UniEvangélica), Anápolis, GO, Brasil

**Keywords:** Poluição por fumaça de tabaco, Pais, Criança

## Abstract

**Objetivo:**

Analisar a percepção dos pais a respeito do tabagismo passivo na saúde de seus filhos.

**Métodos:**

Estudo qualiquantitativo de caráter etnográfico. Buscou-se o ponto de vista e o conhecimento dos pais fumantes ativos quanto à poluição tabagística ambiental e ao tabagismo passivo. Foram incluídos mães e pais fumantes ativos que conviviam diariamente com seus filhos em sete escolas públicas da cidade de Anápolis (GO) no primeiro semestre de 2014. Os pais foram entrevistados em uma sala reservada nas escolas. Procedeu-se à análise descritiva e qualitativa por meio da etnografia.

**Resultados:**

A amostra foi de 58 pais, o tempo médio de tabagismo de 15,3 anos e a quantidade média de cigarros fumados por dia de 20,1. Grande parte (59%) dos pais não sabia o que era poluição tabagística ambiental e 60% disseram saber o que era um fumante passivo. Contudo, quando perguntados a respeito de considerarem seus filhos fumantes passivos, 52% não os consideravam. Observou-se que alguns pais têm conhecimento sobre a influência do tabagismo passivo na saúde de seus filhos. Contudo, a maioria (52%) deles acredita que seus filhos podem não sofrer prejuízo respiratório ou não sabem quais prejuízo são esses.

**Conclusões:**

As crianças analisadas ficavam expostas à poluição tabagística ambiental no domicilio, o que ficou evidente por meio dos dados, do tempo de tabagismo e da média de cigarros fumados por dia. Entretanto, percebeu-se carência no conhecimento dos pais a respeito da poluição tabagística ambiental, do tabagismo passivo e dos males que o cigarro pode causar na saúde dos filhos.

## Introdução

O tabagismo passivo é definido como a inalação da fumaça de derivados do tabaco por indivíduos não fumantes, mas que convivam com fumantes em ambientes fechados. É a terceira causa de morte evitável no mundo, posteriormente ao tabagismo ativo e ao consumo imoderado de álcool. A fumaça dos derivados do tabaco em ambientes fechados é denominada poluição tabagística ambiental (PTA), composta por mais de quatro mil componentes, mais de 40 cancerígenos. De acordo com a Organização Mundial de Saúde (OMS), a PTA torna-se mais prejudicial em ambientes fechados, pois o ar poluído comporta até três vezes mais nicotina e monóxido de carbono e até 50 vezes mais substâncias cancerígenas do que a fumaça que passa pelo filtro do cigarro, inalada pelo fumante ativo.^1^^-^ 3

Dados do Instituto Nacional de Câncer (Inca) de 2011 apontam que o Sistema Único de Saúde (SUS) e a Previdência Social gastam anualmente cerca de R$ 37 milhões com doenças e mortes causadas pelo tabagismo passivo e que o número de óbitos é de cerca de três mil não fumantes por ano. Ainda de acordo com o Inca, a OMS afirma que anualmente cinco milhões de pessoas evoluem para óbito devido às doenças relacionadas ao tabaco e que o tabagismo é a principal causa evitável de morbidade e mortalidade. Estima-se que 1.100 pessoas por dia faleçam devido ao hábito de fumar. Estudos apontam existir cerca de 1,2 bilhão de fumantes no mundo, 24,6 milhões deles somente no Brasil. As estimativas da OMS declaram que 40% das crianças em todo o mundo são expostas à fumaça do tabaco.^4^^-^ 6

O tabagismo prejudica fumantes ativos e passivos e, em longo prazo, produz efeitos deletérios sobre o organismo, como aumento do risco de câncer do trato respiratório, digestório e urinário, do pâncreas, do colo do útero, risco de doenças coronarianas e acidentes vasculares cerebrais. A exposição à PTA encontra-se associada a diversas doenças. Na criança são mais frequentes as infecções do ouvido médio, a redução da função pulmonar e o risco de doenças respiratórias como pneumonia, bronquite e exacerbação da asma. Bebês expostos à PTA têm risco cinco vezes maior de apresentar síndrome da morte súbita infantil, além do risco de doenças pulmonares no primeiro ano de idade.^1^^,^ 7 ^-^ 12 A absorção da fumaça do cigarro por crianças que convivem com pais fumantes em ambientes fechados pode diferir em sua concentração, de acordo com o número de tabagistas no domicílio e a quantidade de cigarros fumados a que a criança é exposta. A OMS relata que os riscos que o tabagismo passivo traz para a saúde são significativos e encontram-se bem constituídos e passíveis de prevenção.^1^^,^ 13

Estudos relatam o conhecimento dos pais a respeito do tabagismo passivo. Entretanto, sua falta ainda é evidente entre as classes socioeconômicas mais baixas. Contudo, mesmo entre aqueles que conhecem os efeitos da PTA, ainda encontram-se pais que expõe seus filhos aos efeitos deletérios do tabagismo intradomiciliar.^13^^,^ 14 Os pais devem compreender que qualquer exposição ao tabaco deve ser considerada um fator de risco para várias doenças. Medidas de controle ao tabagismo que promovam a manutenção de um estilo de vida livre de fumo entre as crianças de todas as idades devem ser implantadas e incentivadas. Dessa forma, este estudo teve como objetivo analisar a percepção dos pais a respeito do tabagismo passivo na saúde de seus filhos.

## Método

Este trabalho apresenta um caráter qualiquantitativo. Para o desenvolvimento do estudo, foi solicitada a autorização das escolas envolvidas. A pesquisa foi aprovada pelo Comitê de Ética e Pesquisa (protocolo: 161.431) do Centro Universitário de Anápolis e foi feita em sete escolas públicas de Anápolis (GO). O contato com os pais foi feito em reuniões escolares, quando foram identificados os fumantes ativos por meio de um diálogo prévio; dessa forma, os participantes foram informados sobre a pesquisa e convidados a participar. Dentre os 356 pais e mães identificados em todas as escolas, 96 declararam-se fumantes e 60 (63%) concordaram em participar e assinaram o Termo de Consentimento Livre Esclarecido (TCLE). Considerou-se apenas um fumante em cada domicílio ou ambos. Após a assinatura os pais responderam às perguntas da entrevista proposta. Foram incluídos na pesquisa somente pais ou mães fumantes ativos, que conviviam diariamente com seus filhos entre seis a 12 anos, e caracterizaram-se 58 pais fumantes. Os dois outros pais foram excluídos por não responderem a todas as perguntas da entrevista. O tamanho da amostra se deu pelo critério de saturação dos dados.

As entrevistas ocorreram mediante um roteiro composto por dados referentes a sexo, idade, tempo de tabagismo, tempo de exposição da criança à PTA, renda familiar e grau de escolaridade. As questões norteadoras foram delineadas para esta pesquisa e composta pelas seguintes perguntas: "Você sabe o que é um fumante passivo?" "Você considera seu filho um fumante passivo?" "Você acha que o seu filho, estando no mesmo ambiente que você, quando você está fumando, pode ter algum prejuízo em sua saúde?" "Sabe relatar qual tipo de prejuízo é esse?" "Você tem conhecimento sobre a influência do tabagismo passivo na saúde respiratória (nos pulmões) do seu filho?" "O que você acha que pode acontecer?" "Você sabe o que é poluição tabagística ambiental?" "Em algum momento já foi orientado a respeito da influência do tabagismo passivo na saúde de seus filhos?"

As entrevistas foram feitas no primeiro semestre de 2014, em uma sala reservada, cedida pela escola, iniciadas e gravadas apenas quando os participantes se sentiam confortáveis para tal. Ao fim da entrevista, os pais foram orientados em relação ao tema, a fim de esclarecer dúvidas de forma a alertá-los quanto aos diversos efeitos e malefícios da PTA e do tabagismo passivo na saúde de seus filhos.

Para transformar as entrevistas em texto, foi usada a transcrição. Nessa etapa, ouviu-se atentamente o conteúdo gravado, foi reproduzida, de modo fiel, a resposta de cada pai e mãe e transformou-se a linguagem oral em escrita, sob a forma de narrativa.

Os dados qualitativos foram analisados por meio da leitura cuidadosa, em busca de captar os aspectos significativos das narrativas e centrando-se nas palavras ou nos sentidos. Extraindo as categorias de análise do fenômeno estudado, foram então selecionadas as frases mais marcantes e semelhantes entre si para formulação de categorias de análise dos resultados de caráter etnográfico. De acordo com Rosa, Lucena e Crossetti, a etnografia vem sendo usada atualmente como método importante para aprimorar fatos relacionados a estilos de vida de indivíduos, considerando os aspectos físicos, culturais, sociais e ambientais e a forma como esses fatores influenciam em suas condições de vida, a partir dos próprios informantes.^15^^,^ 16 Ao se empregar a etnografia, foi possível delinear o caminho para compreender e interpretar a experiência de indivíduos fumantes ativos e sua percepção a respeito dos efeitos do tabagismo passivo na saúde de seus filhos.

A respeito das variáveis, sexo, idade, tempo de tabagismo, números de cigarros fumados por dia, tempo de exposição das crianças à PTA, renda familiar e nível de escolaridade dos pais, foi feita a análise descritiva dos dados, sob forma de média, desvio padrão e frequência relativa e absoluta.

## Resultados

A amostra do estudo foi composta por 58 indivíduos, 66% do sexo feminino e 34% do masculino, todos fumantes ativos com média de 30 anos. Os 58 adultos conviviam com 95 crianças, a idade média foi de 9,2±1,7 anos e 56% eram do sexo feminino.

Quando perguntado o tempo de tabagismo, os pais apresentaram uma média de 15,3 anos. Em relação à quantidade de cigarros fumados por dia e ao tempo de exposição das crianças à fumaça do cigarro, a média foi de 20,1 cigarros e ao redor de 2,8 horas de exposição à PTA por dia. Quando verificado o conhecimento desses pais a respeito da PTA, 59% disseram não ter conhecimento sobre o assunto; porém, 60% desses pais relataram saber o que é um fumante passivo, enquanto 52% disseram não considerar seu filho um fumante passivo. A renda familiar média dos pais girou em torno de R$ 1.389, em relação ao grau de escolaridade, 45% disseram ter frequentado a escola até o ensino fundamental, seguidos de 40% com ensino médio completo, 8% com nível superior e 7% analfabetos.

Os dados gerados com base nas narrativas e descrições constituíram-se no conteúdo de análise. A leitura minuciosa das respostas teve como finalidade captar a presença dos aspectos significativos contidos nas falas dos participantes. Foram escolhidas as frases mais marcantes e semelhantes entre os pais, em cada pergunta.

Quando perguntados "Você sabe o que é fumante passivo?", a maioria dos pais, 60%, disse saber; porém, pela análise das respostas, foi possível observar um nível pobre de conhecimento entre os pais, fato evidenciado pelas falas:

"Sim. Quando eu estou fumando e você está sentindo o cheiro."

"Sim, quando estou fumando e uma pessoa está perto de mim."

"Sim, quem fuma sem filtro de proteção."

"Sim, pessoas que estão perto de quem está fumando."

"Sim, é aquele que está no mesmo ambiente do fumante."

Quando perguntado "Você considera seu filho um fumante passivo?", a maioria dos pais, 52%, afirmou que não considerava. Apenas dois pais justificaram o motivo com as seguintes respostas:

"Não, pois não fumo perto deles."

"Não, porque fumo longe deles."

Observou-se na análise descritiva das respostas à seguinte pergunta "Você tem conhecimento sobre a influência do tabagismo passivo na saúde respiratória (nos pulmões) do seu filho? O que você acha que pode acontecer?" que alguns pais têm conhecimento sobre a influência do tabagismo passivo na saúde respiratória de seus filhos, fato evidenciado pelas falas:

"Sim, acho que pode causar bronquite, não sei, prejudica de alguma forma o pulmão".

"Sim, pode desenvolver vários tipos de doenças no pulmão".

"Vai pro pulmão né, e futuramente causa um problema pulmonar".

"Sim, prejuízo de maneira geral, pode levar a doenças respiratórias".

"Sim, pode ter problemas nos pulmões".

"Acho que tem, mas não sei exatamente o que pode acontecer não".

Porém, observou-se, na análise descritiva, que a maioria dos pais, 59%, relatou não saber o que é PTA e, por meio da análise das respostas da mesma pergunta acima, foi possível confirmar a falta de informação em relação ao tema. A maioria apresentou respostas semelhantes, não acredita que seus filhos poderiam sofrer algum prejuízo respiratório, fato constado nos depoimentos abaixo:

"Não, eles quase não ficam perto de mim e quando eu fumo fico longe".

"Eu e meu marido tentamos não fumar perto dos meninos, então eu acho que eles não vão ter prejuízo".

"Nos pulmões eu acho que não pode acontecer nada. Acho que pode ter outros problemas, mas no pulmão eu acho que não".

"Nos pulmões deles eu acho que não, porque é o meu pulmão que sofre".

"Não, eu acho que só quem vai ter algum prejuízo são eu e meu marido".

"Não, eu vejo só nas caixinhas de cigarro, mas a gente não pensa que pode acontecer".

Quando perguntados se em algum momento já tinham sido orientados a respeito da influência do tabagismo passivo na saúde de seus filhos, a maioria dos pais disse nunca ter recebido qualquer tipo de orientação, com as seguintes respostas semelhantes:

"Não, eu nunca tinha ouvido falar nesse assunto".

"Não, ninguém nunca me falou nada".

"Não, eu nunca tinha ouvido falar do fumo passivo".

"Não. Primeira vez que escuto falar disso".

## Discussão

O tabagismo passivo consiste na exposição secundária à fumaça de cigarro ou outros derivados do tabaco por indivíduos não fumantes, que convivem com fumantes em ambientes fechados. A preocupação do tabagismo passivo em crianças tem efeito em maior escala pelo fato de estarem desenvolvendo seus sistemas corporais, principalmente o sistema respiratório imaturo, que pode ser mais sensível a essa exposição.^1^^,^ 17 ^-^ 19

O ambiente da criança, assim como o convívio com as pessoas adultas, pode exercer influência em seu desenvolvimento. Ao analisar os resultados deste estudo, em relação ao tempo de tabagismo dos pais, encontrou-se uma média de 15,3 anos. Similarmente, uma pesquisa que avaliou a prevalência de sintomas respiratórios em crianças e adolescentes, com 174 pais, apontou que, em relação ao tempo de tabagismo, 46,5% das mães e 57,9% dos pais fumavam havia 14 anos ou mais. Foi demonstrada também forte associação da exposição do tabagismo intradomiciliar como fator de risco para o desenvolvimento e aumento da gravidade da asma em crianças.^18^^-^ 20

Quanto à quantidade de cigarros fumados diariamente, neste estudo os pais fumavam uma média de 20,1 cigarros por dia. Um estudo longitudinal que avaliou o aumento da incidência de asma em crianças de mães fumantes mostrou que crianças cujas mães fumavam mais de meio maço de cigarros por dia, principalmente nos primeiros dois anos de vida, tiveram cerca de duas vezes mais chance de desenvolver asma. Esse fato pode estar associado ao maior contato da mãe com a criança nessa fase da infância.^21^ No presente estudo, a média de tempo de exposição das crianças ao fumo passivo foi de 2,83 horas por dia. Venners et al. avaliaram 1.718 crianças e adolescentes e referiram haver uma discreta relação entre o tabagismo paterno com o declínio na função pulmonar de crianças tabagistas passivas.^22^

A exposição à PTA está associada à elevada morbidade e mortalidade em crianças de baixa idade. A saúde da criança é especialmente vulnerável ao risco dessa exposição, incluindo infecções respiratórias superiores e inferiores. Uma pesquisa que avaliou a exposição da criança à fumaça do tabaco e sua relação com o desenvolvimento da asma apontou que o equilíbrio oxidativo/antioxidativo pendeu fortemente para o lado oxidativo em pré-escolares fumantes passivos, com o desenvolvimento de infecções agudas e crônicas de ouvido, exacerbação da asma, alterações no neurodesenvolvimento, problemas comportamentais e diminuição no rendimento escolar. Os radicais livres originados da fumaça de cigarro são considerados uma causa importante de aterosclerose e câncer e têm a capacidade de induzir direta e indiretamente o estresse oxidativo.^19^^,^ 23

Estudo feito com crianças com sintomas de asma apontaram que 60% dos pais tinham menos de cinco anos de escolaridade. Pesquisa que analisou as diferenças na prevalência de fumantes entre estratos socioeconômicos constatou que entre homens analfabetos ou com menos de quatro anos de estudo formal a prevalência de tabagismo atingiu 48,6%. Outros estudos também constataram que nos domicílios onde os pais tinham menor escolaridade era maior a ocorrência de tabagismo.^24^^-^ 28

Quando avaliada a renda familiar, o presente estudo apresentou uma média de aproximadamente dois salários mínimos. Estudos que consideraram a estratificação da população por faixas de renda e ocupação revelaram consumo mais elevado de cigarros, de duas a três vezes, em grupos com piores condições sociais e econômicas. É importante questionar por que os pais com menor nível socioeconômico são os que mais fumam. O tabagismo pode representar uma resposta ao estresse e às dificuldades associadas com a vida em um ambiente economicamente desprovido.^29^

Quando perguntado aos pais se eles tinham conhecimento sobre a influência do tabagismo passivo na saúde respiratória dos seus filhos, a maioria relatou acreditar que seus filhos não poderiam ter algum prejuízo, nem mesmo na saúde respiratória. Outros pais relataram acreditar que poderão ter apenas problemas respiratórios. Isso mostra que os pais apresentam uma carência de informação a respeito das consequências que a fumaça do cigarro pode gerar na saúde das crianças, frequentemente vitimadas pela ignorância e negligência de adultos fumantes. Estudos comprovam que a fumaça do tabaco gera impactos diretos e indiretos sobre a saúde geral da criança. Algumas delas, filhas de pais fumantes, apresentam fatores associados a dificuldades de aprendizagem e linguagem e problemas de comportamento.^30^No presente estudo alguns pais relataram que não tiveram informação a respeito do tabagismo passivo. Se as informações não chegam às camadas mais pobres e menos informadas de uma sociedade, as crianças com baixo padrão socioeconômico estarão mais vulneráveis aos efeitos deletérios da PTA. Com isso, essas crianças têm mais chance de se tornar fumantes ativas e de adquirir doenças respiratórias e dar continuidade ao ciclo familiar tabagista.^31^

Os esforços para evitar morbidade e mortalidade prematura dependem de programas de prevenção, políticas de proteção contra o tabaco, contra a exposição ao tabaco e eficazes programas de cessação. A cessação contribui para reduzir a carga de doenças provocadas pelo tabagismo, por causa dos benefícios imediatos para a saúde do fumante e das pessoas que convivem com o fumante. No entanto, para muitos fumantes, a cessação permanece uma meta distante. A mudança de comportamento poderá ocorrer quando a motivação para a cessação for alterada, pois há uma ignorância comum sobre a magnitude dos danos do tabaco, em combinação com a tendência dos fumantes a subestimarem seu risco pessoal. A estratégia de abordagem dos pais sendo a promoção da saúde dos filhos expostos à fumaça do tabaco, em vez do risco pessoal, pode ser particularmente eficaz quando o fumante considera que a saúde da criança terá vários benefícios.^32^^,^ 33

Pais e os professores são modelos de comportamento durante a infância. Pais fumantes são fortes exemplos para que seus filhos fumem, o que, além de torná-los tabagistas passivos, pode influenciá-los a um envolvimento com o tabagismo ativo ainda na menor idade e acarretar-lhes sérios problemas de saúde. É importante o desenvolvimento de ações que levem a família e a escola a produzir ações preventivas relacionadas ao consumo de derivados do tabaco.

Vale ressaltar a dificuldade de recrutar pais que se voluntariassem a participar da pesquisa ou que comparecessem às reuniões escolares, da mesma forma que houve uma limitação importante de outras escolas que abrissem as portas para esse tipo de abordagem, o que acabou limitando o número da amostra. Uma fragilidade importante deste estudo é o fato de representar uma população específica e limitada a uma região geográfica (sete escolas públicas de Anápolis, GO), cujos resultados não necessariamente se aplicam a outras regiões do país. Apontam, contudo, a necessidade de mais pesquisas que explorem a percepção dos pais, uma vez que se observou escassez de publicações a respeito do tema.

Conclui-se que há exposição das crianças à poluição tabagística ambiental no domicílio, o que ficou evidente por meio dos dados, do tempo de tabagismo e da média de cigarros fumados por dia. Entretanto, percebeu-se uma carência no conhecimento dos pais a respeito da poluição tabagística ambiental, do tabagismo passivo e dos males que o cigarro pode causar na saúde dos filhos.
